# Carbon-Coated Superparamagnetic Nanoflowers for Biosensors Based on Lateral Flow Immunoassays

**DOI:** 10.3390/bios10080080

**Published:** 2020-07-22

**Authors:** Amanda Moyano, Esther Serrano-Pertierra, María Salvador, José Carlos Martínez-García, Yolanda Piñeiro, Susana Yañez-Vilar, Manuel Gónzalez-Gómez, José Rivas, Montserrat Rivas, M. Carmen Blanco-López

**Affiliations:** 1 Department of Physical and Analytical Chemistry & Institute of Biotechnology of Asturias, University of Oviedo, c/Julián Clavería 8, 33006 Oviedo, Spain; moyanoamanda@uniovi.es (A.M.); serranoesther@uniovi.es (E.S.-P.); 2 Department of Physics & IUTA, University of Oviedo, Campus de Viesques, 33204 Gijón, Spain; salvadormaria@uniovi.es (M.S.); jcmg@uniovi.es (J.C.M.-G.); rivas@uniovi.es (M.R.); 3 Department of Applied Physics, University of Santiago de Compostela, Campus Vida, 15782 Santiago de Compostela, Spain; yolanda.fayoly@gmail.com (Y.P.); susana.yanez@usc.es (S.Y.-V.); manuelantonio.gonzalez@usc.es (M.G.-G.); jose.rivas@usc.es (J.R.)

**Keywords:** superparamagnetic iron oxide nanoflowers, lateral flow immunoassays, biosensor, extracellular vesicles, exosomes

## Abstract

Superparamagnetic iron oxide nanoflowers coated by a black carbon layer (Fe_3_O_4_@C) were studied as labels in lateral flow immunoassays. They were synthesized by a one-pot solvothermal route, and they were characterized (size, morphology, chemical composition, and magnetic properties). They consist of several superparamagnetic cores embedded in a carbon coating holding carboxylic groups adequate for bioconjugation. Their multi-core structure is especially efficient for magnetic separation while keeping suitable magnetic properties and appropriate size for immunoassay reporters. Their functionality was tested with a model system based on the biotin–neutravidin interaction. For this, the nanoparticles were conjugated to neutravidin using the carbodiimide chemistry, and the lateral flow immunoassay was carried out with a biotin test line. Quantification was achieved with both an inductive magnetic sensor and a reflectance reader. In order to further investigate the quantifying capacity of the Fe_3_O_4_@C nanoflowers, the magnetic lateral flow immunoassay was tested as a detection system for extracellular vesicles (EVs), a novel source of biomarkers with interest for liquid biopsy. A clear correlation between the extracellular vesicle concentration and the signal proved the potential of the nanoflowers as quantifying labels. The limit of detection in a rapid test for EVs was lower than the values reported before for other magnetic nanoparticle labels in the working range 0–3 × 10^7^ EVs/μL. The method showed a reproducibility (RSD) of 3% (n = 3). The lateral flow immunoassay (LFIA) rapid test developed in this work yielded to satisfactory results for EVs quantification by using a precipitation kit and also directly in plasma samples. Besides, these Fe_3_O_4_@C nanoparticles are easy to concentrate by means of a magnet, and this feature makes them promising candidates to further reduce the limit of detection.

## 1. Introduction

The small size and the unique properties of magnetic nanoparticles have aroused considerable interest in the field of nanomedicine. In fact, they are currently being studied for biomedical applications such as hyperthermia, drug delivery, biosensors, and imaging [1,2]. Superparamagnetic nanoparticles (SPM NPs) are especially attractive due to their large saturation magnetization and initial magnetic permeability [3]. One of the most interesting features of SPM NPs is the possibility to be attracted by magnetic field gradient and then redispersed when the field is removed. This enables remote manipulation at immunoseparations for analytical determinations and other customized applications.

Magnetite and maghemite are the preferred crystal structures of iron oxide superparamagnetic nanoparticles due to their good magnetic response, biocompatibility, facile synthesis, and low-cost production. In addition, they can be modified with different surface coatings for electrosteric stabilization to prevent their uncontrolled agglomeration in solution [4]. Core/shell superparamagnetic nanoparticles are considered as a superparamagnetic core coated with other materials such as polymers, gold, silver, fatty acids, or surfactants, which increase colloidal stability and biocompatibility, preventing the oxidation of the iron oxide core and enhancing chemical versatility by linking functional groups [5]. A chemical versatile shell is desirable for the conjugation of bioreceptors such as antibodies, nucleotides, or peptides, which are used for molecular recognition of specific targets at analytical tests and devices [6].

Carbon coatings have several advantages as capping agents, since they are chemically stable, cheap, and light and allow an easy encapsulation of iron oxide cores [7,8,9]. Additionally, carbon films include carboxylic groups enabling the bioconjugation of nanoparticles with receptors for molecular recognition [10,11]. Carbon nanoparticles and other carbon nanomaterials have been reported as novel labels in lateral flow due to their advantages such as its strong color and its high contrast against the white background of nitrocellulose membrane [12,13]. Moreover, the limit of detection reached for these labels have been demonstrated to be better than those obtained with gold nanoparticles [14,15]. Therefore, the carbon coating can develop a strong visual signal as other carbon materials, and this could be advantageous when the nanoparticles are used in biosensors based on immunoassays.

In recent years, biosensors based on superparamagnetic nanoparticles have received considerable attention due to magnetic nanoparticles properties mentioned above, because they enhance the analytical figures of merit of biosensors such as electrochemical, optical, and piezoelectric sensors [16]. The magnetic nanoparticles are generally used to attract the analyte towards the detection surface by an external magnetic field. Sometimes, they are even integrated into the sensor transducer. Numerous biosensors based on superparamagnetic nanoparticles have been reported by using electrochemical [17,18,19], optical [20,21], piezoelectric [22,23], and magnetic [24,25,26,27] transducers.

Lateral flow immunoassay (LFIA) is a rapid test that meets the requirements for point of care (POC) testing. It is simple and provides results within a short time without the necessity of centralized laboratory at low cost [28]. The tests consist of different cellulosic elements (sample pad, conjugate pad, nitrocellulose membrane, and adsorption pad), which are assembled on a plastic backing to get robustness. Biorecognition elements are immobilized on the nitrocellulose membrane displaying lines (generally control and test lines), which become active upon flow of the liquid sample that contains the analyte of interest and nanoparticles. Generally, this kind of test relies on a visual signal, providing a yes/no response by means of colored nanoparticles such as gold or latex [29]. Magnetic nanoparticles are key players for the development of quantitative LFIA in combination with external magnetic transductors [30]. Additionally, magnetic nanoparticles have been used as colorimetric labels detected by naked-eye and optical readers due to their dark brown color easily distinguishable on white nitrocellulose membranes [31,32,33]. To date, several LFIA based on magnetic nanoparticles have been reported to detect and quantify human immunodeficiency virus (HIV) [34], allergen parvalbumin [35], and *Bacillus anthracis* spores [36,37] by means of a magnetic assays readers (MAR) system. The magnetic measurements require the excitation of the magnetic nanoparticles used as labels in the immunoassay by an oscillating magnetic field in order to quantify the magnetization of the nanoparticles. Moreover, magnetic nanoparticles can be used in order to purify and enrich analytes, enhancing the limits of detections and selectivity of conventional LFIA [38,39,40,41].

Our research group developed a novel magnetic sensor coupled to LFIA to quantify superparamagnetic nanoparticles immobilized at test and control lines. We used it to determine prostate specific antigen concentrations in the clinical range of interest by using a sandwich format [42] and histamine in wine following a competitive immunoassay [43]. For those studies, we used 10–12 nm size magnetic nanoparticles, which are stable in solution due to their electrostatic repulsion. The sensor is simpler compared with others, because the external field is not required. However, the superparamagnetism of the nanoparticles should be carefully optimized in order to produce the desired increase of impedance on a radio frequency (RF) current-carrying copper conductor [44,45]. This impedance change is directly proportional to the number of magnetic nanoparticles. Thus, this device allows an indirect detection of analyte of interest thanks to magnetic reporters.

The aim of this work was to study the use of iron oxide nanoflowers coated by a black carbon layer (Fe_3_O_4_@C) as labels for LFIA. The nanoflower structure consists of several superparamagnetic cores embedded with an external carbon surface. This core-shell structure was designed aiming its use at both immunoseparation and detection; multiple cores are suitable for immunoisolation, and the carbon coating provides a strong optical signal, keeping the magnetic properties unaltered.

In order to test their applicability to LFIA, we firstly studied these Fe_3_O_4_@C nanoparticles with a model affinity molecular recognition system. With this aim, we bioconjugated the particles to neutravidin and tested them against biotin printed across the membrane. Then, the Fe_3_O_4_@C nanoparticles were applied to an immunoassay for extracellular vesicles (EVs). EVs are nanovesicles produced by all cells via endocytosis processes. They carry proteins and nucleic acids from the original cell and therefore can be used to get molecular information about their parent cells. They are attracting a lot of attention in recent years as a source of non-invasive biomarkers for liquid biopsy. The bottleneck at EV research that is limiting industrial and clinical translations is the current isolation from biological fluids and the development of simple quantification methods. Our research group developed LFIA for EVs based on tetraspanin recognition [46,47]. In this work, as proof of concept, we tested the potential of the Fe_3_O_4_@C nanoparticles to EVs separation and quantification.

## 2. Materials and Methods

### 2.1. Chemicals and Instruments

Ferrocene (Fe(C_5_H_5_)_2_, 98%), hydrogen peroxide (H_2_O_2_, 30%), acetone (C_3_H_6_O, 99.9%), bovine serum albumin (BSA), 1-ethyl-3-[3dimethylaminopropyl]-carbodiimide hydrochloride (EDC), and 2-(*N*-morpholino)ethanesulfonic acid (MES) were of analytical reagent grade purchased from Aldrich (Madrid, Spain) and were used without further purification. NeutrAvidin protein was obtained from Thermo Fischer Scientific (Waltham, MA, USA).

For biotin–neutravidin tests, glass fiber membrane (GFCP001000) used as sample pad and backing cards (HF000MC100) were purchased from Millipore (Darmstadt, Germany). Other materials used were nitrocellulose membranes (UniSart CN95, Sartorius, Spain) and absorbent pads (Whatman, Madrid, Spain). The sample buffer consisted of 10 mM phosphate buffer (PB) pH 7.4 with 0.5% Tween-20 and 1% BSA.

For EVs tests, nitrocellulose membranes (HF07504XSS) were purchased from Millipore (Germany). Other materials used for the preparation of strips were similar to the biotin-neutravidin test. Based on previous results, the sample buffer consisted of 10 mM HEPES pH 7.4 with 0.5% Tween-20 and 1% BSA. HEPES was purchased from Fisher Scientific (Madrid, Spain). Anti-tetraspanin antibodies anti-CD9 and anti-CD63 were provided by Immunostep (Salamanca, Spain). Anti-mouse IgG was purchased from Sigma-Aldrich (Spain). Lyophilized commercial exosomes purified from plasma (HBM-PEP) of healthy donors were purchased from HansaBioMed (Tallinn, Estonia).

In order to dispense the control and the detection lines, an IsoFlow reagent dispensing system (Imagene Technology, Lebanon, NH, USA) was used with a dispense rate of 0.100 μL/mm. A guillotine Fellowes Gamma (Madrid, Spain) was used to cut the strips. For quantification at the test line by reflectance measurements, a portable strip reader ESE Quant LR3 lateral flow system (Qiagen Inc., GmbH, Hilden, Germany) was used.

### 2.2. Synthesis and Characterization of Carbon-Coated Nanoflowers

#### 2.2.1. Synthesis

The synthesis was carried out following a one-pot solvothermal method as previously reported by Wang [48]. Typically, ferrocene (m = 0.3 g) was dissolved in acetone (V = 25 mL) under vigorous magnetic stirring for 30 min; then, hydrogen peroxide (V = 1.5 mL) was slowly added into the above mixture solution and vigorously stirred for another 30 min. This precursor solution was transferred to the Teflon-lined stainless-steel autoclave (Parr Instrument, Illinois, IL, USA) with a total volume of 45.0 mL and heated to 210 °C for 96 h. Finally, the autoclave was cooled to room temperature, the reaction products were magnetically collected, and the supernatant was discarded. The precipitates were washed with acetone four times, and again, the products were magnetically separated to eliminate the acetone and were repeatedly washed with water.

#### 2.2.2. Characterization

Hysteresis loops were recorded with a vibrating sample magnetometer (VSM, DMS, Lowell, MA, USA) at room temperature and under external magnetic fields from −10,000 to 10,000 Oe.

Room temperature X-ray diffraction (XRD) patterns of powder samples were obtained with a Philips PW1710 diffractometer (Panalytical, Callo End, UK) with a Cu Kα radiation (λ = 1.54186 Å) between 10° and 80° with steps of 0.02° and 10 s/step.

The composition was analyzed with a TGA Perkin Elmer model 7 (Perkin, Waltham, MA, USA).

Fourier transform infrared (FTIR) spectra were performed in a Thermo Nicolet Nexus spectrometer (Thermo Fisher Scientific, Madrid, Spain) using the attenuated total reflectance (ATR) method from 4000 to 400 cm^−1^.

Scanning electron microscopy (SEM) with a Zeiss FE-SEM ULTRA Plus (5 kV) microscope (Zeiss, Oberkochen, Germany) and transmission electron microscopy (TEM) with a JEOL JEM-1011 microscope (100 kV) were employed to study the morphology.

### 2.3. Bioconjugation of Superparamagnetic Iron Oxide Nanoflowers Coated by Fe_3_O_4_@C

#### 2.3.1. Functionalization

Fe_3_O_4_@C with carboxyl functional groups were functionalized using neutravidin to test their performance as label in immunoassays through the neutravidin–biotin interaction. Firstly, 100 µL of nanoflowers were mixed with 100 µL of neutravidin (different concentrations were studied) and 20 µL of EDC (1 mg/mL in MES 1 mM, pH 6.00) under continuous sonication for one hour. Then, 20 µL of EDC were added one hour and two hours after under sonication. After the last addition of EDC, the mixture was sonicated for 10 min.

The protocol to cover nanoflowers with neutravidin was adapted to bioconjugate the nanoflowers to antitetraspanin antibodies against EVs. Firstly, 50 µL of nanoflowers were mixed with 50 µL of anti-CD63 (1 mg/mL) and 20 µL of EDC (1 mg/mL in MES 1 mM, pH 6.00) under continuous sonication for one hour. Then, 20 µL of EDC were added one hour and two hours after under sonication. After the last addition of EDC, the mixture was sonicated for 10 min.

#### 2.3.2. Characterization of Nanoparticles Conjugates by Dynamic Light Scattering

A Zetasizer Nano ZS ZEN3600 (Malvern Instruments, Malvern, UK) equipped with a solid-state He–Ne laser (λ = 633 nm) was used to measure size distribution and ζ-potential. In order to monitor the conjugation process, 30 measurements of the backscattered (173°) intensity were carried out at 25 °C and averaged. For data analysis, Zetasizer software version 7.03 was used.

### 2.4. Enrichment and Quantification of EV from Real Samples

Extracellular vesicles derived from human plasma samples were isolated using ExoQuick^TM^ precipitation reagent (System Biosciences, Palo Alto, CA, USA), according to the manufacturer’s instructions. Freshly isolated EVs were analyzed using a NanoSight LM10 instrument (Malvern, Worcestershire, UK) and NTA 3.1 software at Nanovex Biotechnologies S.L (Asturias, Spain).

### 2.5. Lateral Flow Assays

#### 2.5.1. Preparation of the Strips

The immunoassay was based on a dipstick format. The strips consist of four parts: sample pad, nitrocellulose membrane, absorbent pad, and backing plastic card. The first step was to incorporate the nitrocellulose membrane into the backing plastic card to get robustness. Then, for biotin–neutravidin affinity test, a biotin-BSA test line was immobilized on the membrane with a concentration of 1 mg/mL. An IsoFlow dispenser at a rate of 0.100 µL/mm was employed.

For EVs tests, two lines of antibodies were immobilized across the nitrocellulose strip: (i) the test line gave us the result of the analysis following a sandwich format and (ii) the control line was used to validate the strip indicating that the liquid sample flowed adequately along the strip. Both lines were applied by the IsoFlow dispenser at a rate of 0.100 μL/mm with 1 mg/mL concentration of anti-CD9 and anti-IgG for test line and control line, respectively.

The nitrocellulose membrane after dispensing was kept for 20 min at 37 °C. Finally, the absorbent pad and the sample pad were stuck onto the backing card overlapping them 2 mm. Finally, individual strips of 5 mm were cut. For storage, strips were kept at room temperature and preserved with desiccant bags to avoid moisture.

#### 2.5.2. Magnetic Quantification

Quantification of the test line in the LFIAs was provided by a Scanning MagnetoInductive Sensor (SMISensor) specifically designed in-house for this task. The sensing head consists of a double copper line printed on a rigid insulating substrate across which an alternating current is continuously flowing. A precision impedance analyzer (Agilent 4294A, Agilent Technologies, Madrid, Spain) was used to monitor the magnitude and the phase of the sensing head impedance. For this purpose, 16048G test leads and a 500 mV/40 MHz excitation voltage were used. The test lines on the strips were scanned laterally over the sensing head by a micro-positioner producing an increase of the impedance of the circuit due to the large magnetic permeability of the superparamagnetic particles present on it. This variation proved to be directly proportional to number of nanoparticles at the test line in previous studies. To account for all the particles in the test line, the signal was integrated across the position. For further information, please, see Supplementary Materials.

#### 2.5.3. Optical Measurements

In order to quantify the color intensity of the test line by reflectance measurements, a portable strip reader ESE-Quant LR3 lateral flow system (Qiagen Inc., Hilden, Germany) was used.

#### 2.5.4. Characterization of the Strip by SEM

The morphology of the strips was characterized by SEM in a Zeiss FE-SEM ULTRA Plus (5 kV) microscope (Zeiss, Oberkochen, Germany).

## 3. Results and Discussion

### 3.1. Characterization of the Carbon-Coated Nanoflowers before Bioconjugation

**Figure 1:**
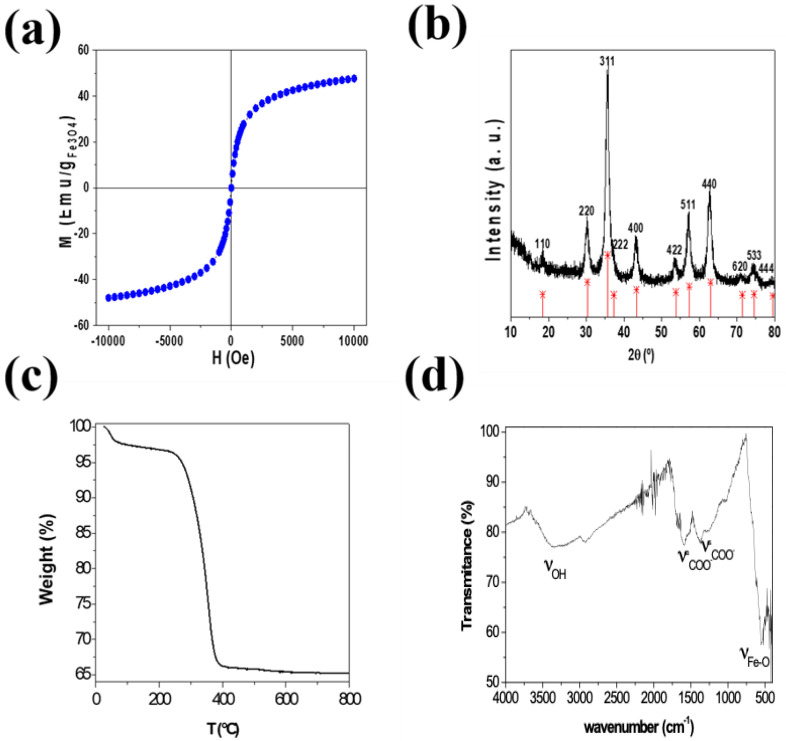
(**a**) Hysteresis loops of core/shell structure of magnetite/carbon performed at room temperature with a vibrating sample magnetometer (VSM) (−10, +10) KOe. (**b**) X-ray diffraction (XRD) pattern of the core/shell structure of magnetite/carbon colloidal nanoparticles compared to the XRD pattern of magnetite from JCPDS 19-0629 data base (**c**) thermogravimetric analysis (TGA) curves of the core/shell structure of magnetite/carbon colloidal nanoparticles. (**d**) FT-IR.

After synthesis, carbon-coated nanoflowers were characterized using various analytical techniques: VSM, X-ray diffraction, ζ potential, thermogravimetric analysis (TGA), FTIR, SEM, and TEM (Figure 1).

Magnetic characterization of the dried samples performed with a VSM at room temperature showed a magnetization normalized to the content of magnetite with a saturation at 10 KOe around 50 emu. This indicated superparamagnetic behavior (Figure 1a), in concordance with the dominant surface effects in small nanoparticles, for which the dead magnetic layer significantly decreased the magnetization. Moreover, negligible coercivity and absent remanence were observed. This can be ascribed to the superparamagnetic behavior of small magnetite nanoparticles.

Figure 1b shows X-ray diffraction spectra of the carbon coated multicore NP shown together with the theoretical diffraction peaks of magnetite (JCPDS card No. 19-0629) [49]. It can be seen that both the location and the relative intensity of m-Fe_3_O_4_@C nanoparticles coincided with the main theoretical 111, 220, 311, 400, 422, 511, 440 magnetite reflections (red lines in Figure 1b). This confirmed that magnetite was the crystalline phase of iron oxide present in the sample. To obtain the crystallite size, Scherrer formula [50] was applied to main reflection peak 311, providing an average Dhkl = 16.3 nm and σ = 2.8 nm. Moreover, on low diffraction angles, a broad band corresponding to the amorphous carbon coating shell could be seen.

The surface charge of Fe_3_O_4_@C nanoparticles was negative with a ζ potential value of -32 mV and a rate of magnetite/total mass of 0.651% (Wmagnetite/Wsample) (Figure 1c). The ζ potential value confirms the stability of Fe_3_O_4_@C nanoparticles in water suspension.

Figure 1d shows the FT-IR spectra of Fe_3_O_4_@C nanoparticles. The peak observed around 550 cm^−1^ was characteristic of Fe-O vibrations [51]. Additionally, a large band around 3400 cm^−1^ was also observed due to the -OH groups adsorbed on the nanoparticle surface. Besides, two peaks appeared around 1595 and 1384 cm^−1^, which corresponded to the asymmetric and the symmetric stretching vibrations of COO- groups.

**Figure 2:**
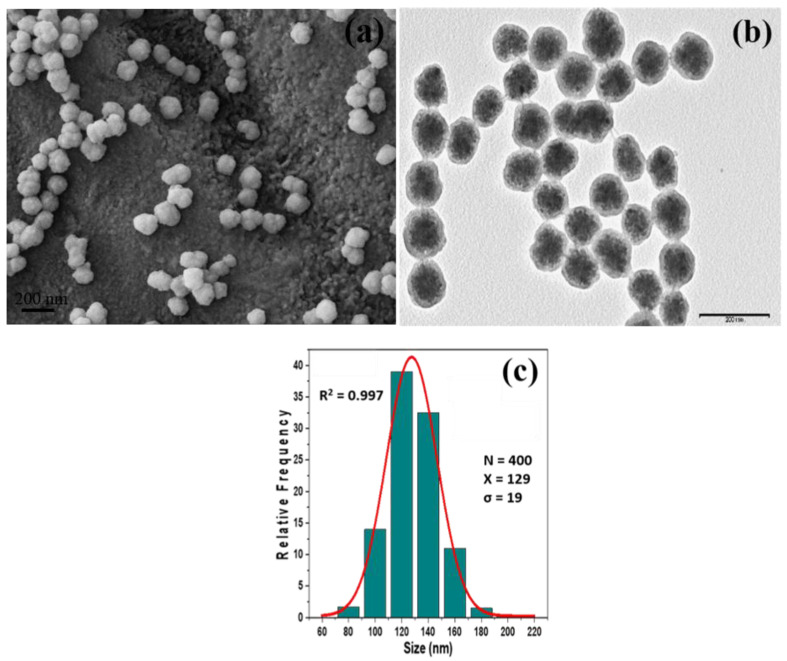
(**a**) SEM and (**b**) TEM images of the core/shell structure of magnetite/carbon colloidal NPs. (**c**) Analysis of size distribution obtained from (**b**).

The morphology of the Fe_3_O_4_@C nanoparticles was characterized by scanning electron microscopy (SEM) and by transmission electron microscopy (TEM), as shown in Figure 2a,b respectively. It can be seen (Figure 2a) that the obtained particles had a nearly spherical shape and uniform size with a regular core@shell structure where the carbon coating homogeneously encapsulated dozens of magnetite nanocrystals with a size 10–20 nm (Figure 2b). From TEM images, distribution of sizes was analyzed showing a mono-modal histogram with slight polydispersity, with an average diameter of 129 nm ± 19 nm (Figure 2c).

### 3.2. Study of Neutravidin Concentration during the Bioconjugation Process

**Figure 3:**
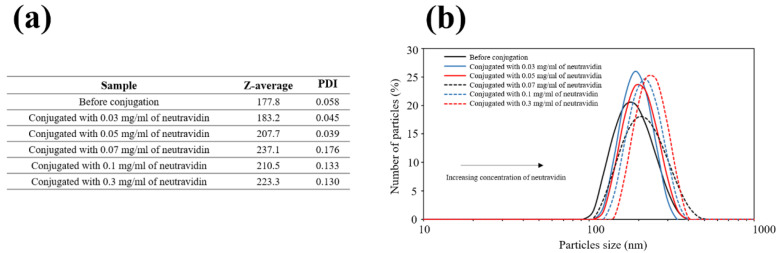
(**a**) Results obtained by dynamic light scattering (DLS) (*Z*-average and polydispersity index) for study performed with neutravidin. (**b**) Hydrodynamic size distribution profiles of Fe_3_O_4_@C nanoparticles before (solid black line) and after conjugation with neutravidin concentrations of 0.3 mg/mL (solid red line), 0.1 mg/mL (solid blue line), 0.07 mg/mL (dashed black line), 0.05 mg/mL (dashed red line), and 0.03 mg/mL (dashed blue line) of neutravidin.

Increasing neutravidin concentrations were used to coat the nanoflowers: 0.03, 0.05, 0.07, 0.1, and 0.3 mg/mL. Phosphate buffer was used as diluent to prepare the neutravidin standards. Dynamic light scattering (DLS) measurements were carried out to compare nanoparticles hydrodynamic size before and after conjugation reaction. The hydrodynamic diameter of nanoparticles before conjugation was 178 nm (polydispersity index 0.058). The Figure 3a shows the values for *Z*-average of the hydrodynamic sizes for the different concentration of neutravidin used during the conjugation process. The hydrodynamic diameter of nanoparticles was higher when the concentration of neutravidin increased (Figure 3b). The bioconjugation process was confirmed by the increase of the hydrodynamic diameter of nanoparticles before and after conjugation. The mean size of neutravidin is around 2–3 nm (60 kDa). Therefore, considering the neutravidin size, it can be confirmed that this protein was bound to nanoparticles surface though the carboxyl functional groups available. The *Z*-average of the hydrodynamic sizes increased directly with the concentration of neutravidin. This could be because more than one molecule of neutravidin can be bound to a nanoparticle through their multiple functional groups on their surface.

### 3.3. Lateral Flow Assay Procedure

**Figure 4:**
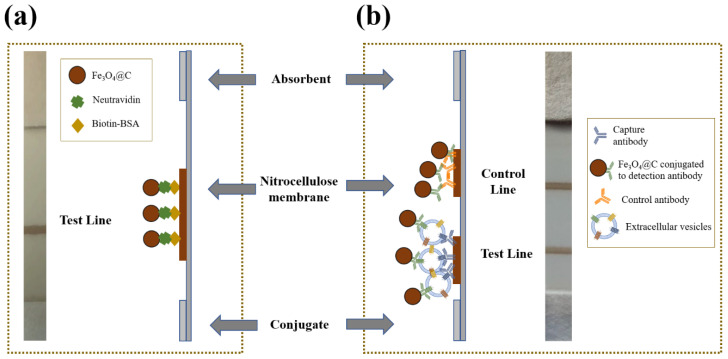
(**a**) Photography (front view) and schematic illustration of the biotin–neutravidin affinity test (side view). (**b**) Photography (front view) and schematic representation of the lateral flow immunoassay for extracellular vesicles (side view).

A biotin–neutravidin affinity test (Figura 4a) and a lateral flow immunoassay for extracellular vesicles (Figure 4b) were developed.

#### 3.3.1. Biotin-Neutravidin Affinity Test

The biotin-streptavidin/neutravidin/avidin system has been widely used in immunoassays because it is a powerful non-covalent interaction with high specificity and strong affinity [52,53]. Streptavidin, neutravidin, and avidin are molecules that contain four binding sites with an extraordinarily high affinity for biotin. This system is very attractive for biosensing because of its amplification capability [54,55,56]. In addition, biotin can be easily covalently bonded to proteins such as antibodies, nucleotides, and enzymes, enabling a strong binding between biotinylated proteins and streptavidin, neutravidin, or avidin. In this case, the biotin–neutravidin system was used as model system for affinity interactions as a first step to study the feasibility to bioconjugate these nanoparticles.

In order to test the suitability of the NP for LFIA, 20 µL of suspensions with different concentration of neutravidin and 80 µL of running buffer were transferred into a microtube. The sample pad was introduced into the mixture, and the buffer started to flow through the strip by capillary action. Only the nanoflowers coated with neutravidin were retained at the biotin-BSA line, as shown in Figure 4. A simplified schematic representation of biotin–neutravidin interaction in LFIA is shown in Figure 4.

#### 3.3.2. Magnetic and Optical Quantification for Biotin-Neutravidin Test

**Figure 5:**
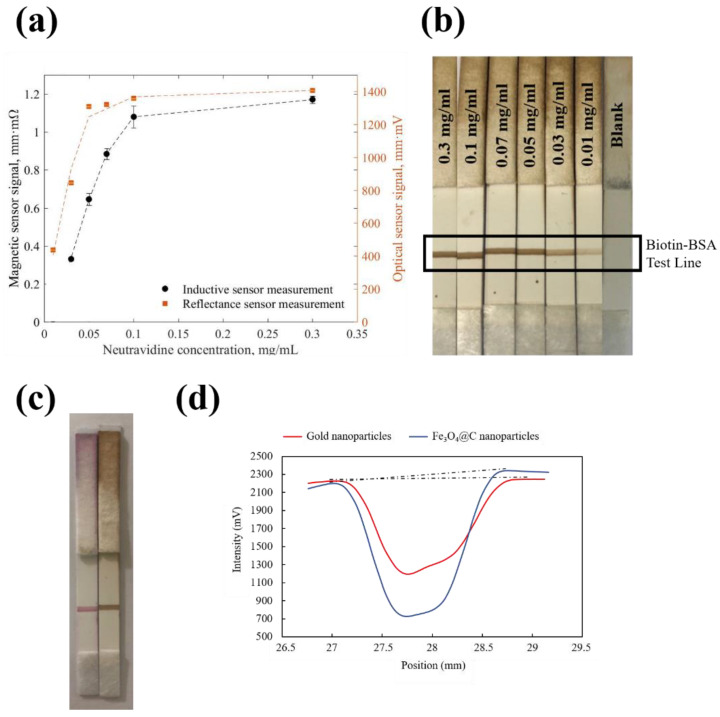
(**a**) Magnetic and optical signals as function of the concentration of neutravidin. (**b**) Representative example of results obtained in the strips for the different concentrations of neutravidin. (**c**) Comparison of different labels: gold nanoparticles (left) and Fe_3_O_4_@C nanoparticles (right). (**d**) Intensity profiles measured using ESEQuant reader for gold (red line) and Fe_3_O_4_@C (blue line) nanoparticles.

The test line, once dried, was analyzed by reflectance and magnetic measurements. Figure 5a shows the results for both measurements. The optical and the magnetic signals increased with neutravidin concentration until saturation was reached. The saturation was produced due to the depletion of free biotin molecules at the test line, thus the nanoflowers coated with neutravidin were not retained. The optical and the magnetic methods showed a reproducibility (RSD) of 1% (n = 3) for both cases. Figure 5b shows a representative example of strips for an increase in neutravidin concentration. Both curves followed the same trend, achieving a slightly better fit for the magnetic one. The signal corresponding to nanoparticles coated with 0.01 mg/mL of neutravidin was difficult to distinguish from the blank at the inductive sensor. However, the optical signal was significantly different from the blank, as Figure 5b shows. For the blank, Fe_3_O_4_@C nanoparticles without neutravidin coating were assayed in order to test that bare nanoparticles were not attached to biotin-BSA test line. The Figure 5b confirms that there were not non-specific interactions between bare Fe_3_O_4_@C nanoparticles and biotin-BSA test line. A higher concentration of neutravidin (0.03 mg/mL) on the nanoparticles could be easily detected by both magnetic and optical instruments.

Colloidal gold was used as label at the same immunoassay (biotin-neutravidin interaction test) to compare the results with those of Fe_3_O_4_@C nanoparticles. Colloidal nanoparticles have been widely used as labels in LFIA, the 40 nm size being the most popular choice for this kind of assay due to their higher sensitivity [57]. Gold nanoparticles were conjugated to neutravidin by passive binding [58]. The optimal concentration of neutravidin to stabilize the colloidal gold was found through a titration assay as described in reference [58]. The protocol for the titration was executed as described elsewhere [58]. The results indicated that 0.4 mg/mL of neutravidin was the lower concentration needed for the conjugation with gold nanoparticles (data no shown). To perform the comparison, Fe_3_O_4_@C nanoparticles were conjugated to 0.3 mg/mL of neutravidin, because at this concentration, the optical signal has reached the saturation (Figure 5a), and therefore the intensity of this signal should be similar to that obtained by 0.4 mg/mL of neutravidin. Therefore, the concentrations chosen were comparable for both labels.

The test was carried out using the same protocol described above, and the optical reader was used to analyze the signals. Figure 5c shows that naked-eye signals for gold and Fe_3_O_4_@C nanoparticles at the test line apparently looked equivalent. However, when the test lines were quantified by optical reader, the results show that the nanoflowers displayed a significantly stronger optical signal (1405.9 mm·mV) compared with gold nanoparticles (937.3 mm·mV). Figure 5d shows the intensity profiles measured using ESEQuant reader for gold nanoparticles (red line) and Fe_3_O_4_@C nanoparticles (blue line). The intensity profiles were based on reflectance measurements. The ESEQuant reader scanned the test line by moving the light source over the strip. The reader was adjusted so that the light reflected from the strip was collected by a confocal detector and registered as intensity (mV). When the incident beam passed across the test line, the reflected light decreased compared with the light reflected from the membrane (base line). This was because the nanoparticles at the test line absorbed light, and this effect reduced light intensity. This was shown as a negative peak (Figure 5d). Hence, the recorded graph represents intensity (mV) versus position (mm). The result of reflectance measurements for gold and Fe_3_O_4_@C nanoparticles were obtained by integration of negative peak area (Figure 5d, dashed black lines). Absorption and reflection of the light were proportional to the overall intensity of the nanoparticles of the test line. Therefore, the results indicate that the density of Fe_3_O_4_@C nanoparticles was higher that the density of gold nanoparticles at the biotin-BSA test line for a similar concentration of neutravidin.

#### 3.3.3. Characterization of the Strip by SEM

**Figure 6:**
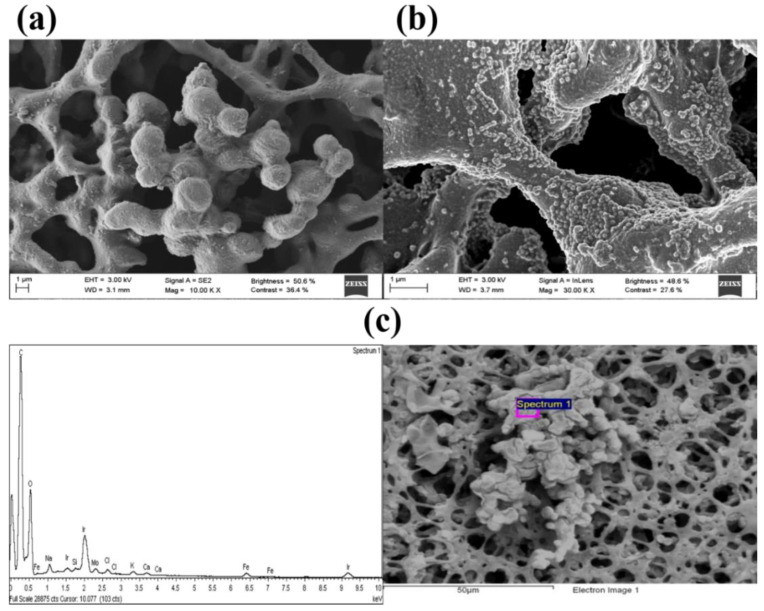
(**a**) SEM images of the cellulose membrane and (**b**) Fe_3_O_4_@C nanoparticle agglomerates attached to the cellulose network at the test line. The strips were examined after use in the detection device. (**c**) energy-dispersive X-ray spectroscopy (EDS) spectrum obtained from one particular location in a SEM image of the strips with presence of Fe_3_O_4_@C nanoparticles.

Morphological characterization of the strips was carried out by SEM in order to observe the distribution of the Fe_3_O_4_@C nanoparticles after using the strip in the detection device. Figure 6a shows an image of the porous structure of a non-colored part of the strip. Spheres with size between 3–5 µm embedded in a network of fibers were clearly observed in the region corresponding to the test line (Figure 6b). We tried several membranes with different pore sizes to let Fe_3_O_4_@C nanoparticles flow along membrane until the end of the strip. It could be observed that the pores of this cellulose network were large enough to allow the diffusion of the Fe_3_O_4_@C nanoparticles through the membrane (Figure 6b).

An energy-dispersive X-ray spectroscopy (EDS) analysis was also carried out in order to confirm that these spherical nanoparticles corresponded to Fe_3_O_4_@C nanoparticles agglomerates. The EDS spectrum showed the presence of Fe on the analyzed area (Figure 6c). With this analysis, we confirmed the presence of Fe_3_O_4_@C nanoparticles on the test line.

#### 3.3.4. Applications of Fe_3_O_4_@C Nanoparticles for Detection of EVs by LFIA

Finally, as proof of concept, we developed a lateral flow immunoassay for EVs by using these nanoflowers as label. The immunoassay relies on sandwich format developed in our research group [46,47]. Anti-CD9 and anti-CD63 were used as capture and detection antibodies, respectively. Capture antibody was immobilized on the nitrocellulose membrane at the test line, and detection antibody was conjugated to the nanoflower surfaces. Figure 4b shows a schematic illustration of the immunoassay for EVs. Commercial standard exosomes were used to study these magnetic nanoparticles as labels and to evaluate the magnetic signal in this application.

In order to carry out the test, different concentrations of standard commercial exosomes (6.00 × 10^6^, 1.50 × 10^7^, 2.10 × 10^7^, 3.00 × 10^7^, 6.00 × 10^7^, 1.05 × 10^8^ EVs/µL) were added into a microtube that contained 10 µL nanoflowers coated with anti-CD63 and buffer until a final volume of 100 µL. HEPES was used to dilute the standard commercial exosomes.

**Figure 7:**
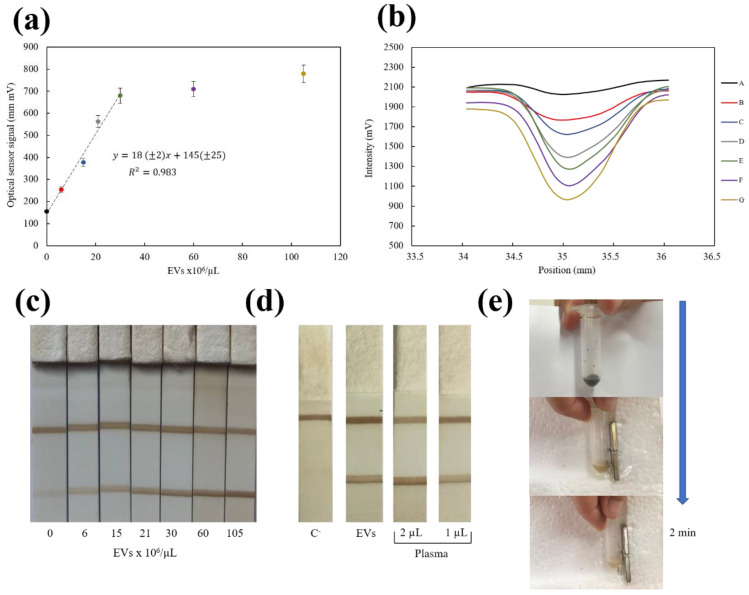
(**a**) Optical calibration curve for extracellular vesicles (EVs). (**b**) Intensity profiles measured using ESEQuant reader for different concentration of EVs: blank (A), 6.00 × 10^6^ (B), 1.50 × 10^7^ (C), 2.10 × 10^7^ (D), 3.00 × 10^7^ (E), 6.00 × 10^7^ (F), 1.05 × 10^8^ (G) EVs/µL (**c**) Representative example of results obtained in the strips with different concentrations of standard EVs. (**d**) Representative strips of lateral flow immunoassay (LFIA) tested with real samples: negative control (C-), EVs isolated from plasma sample using ExoQuick (EVs), and plasma. (**e**) Separation of Fe_3_O_4_@C nanoparticles using a conventional magnet.

The strips were analyzed at both the magnetic sensor and the optical reader, but quantification was possible only with the optical reader. Previous results were based on the plain biotin–neutravidin interaction, but the assay used for the EVs detection was more complex; it involved a capture antibody of bulky EVs at the test line and Fe_3_O_4_@C bioconjugated antibody for detection. The optical signal corresponding to the highest concentration of EVs was 779 mm·mV (Figure 7). If we look at Figure 5a, the corresponding value at the magnetic sensor curve for that concentration would be below the quantification possibilities of this device. Further work is in progress to optimize the microelectronics for amplification.

Nevertheless, a direct relationship between the concentration of exosomes and the optical signal was found (Figure 7). Figure 7b shows the intensity profiles obtained by commercial optical reader for different concentration of EVs. Reflectance peak area increased proportionally with EVs concentration since the density of Fe_3_O_4_@C nanoparticles at the test line was higher. The limit of detection (LOD) achieved was 4 × 10^6^ EVs/µL (calculated with the 3 S_B_/m criterium), and the method showed a reproducibility (RSD) of 3% (n = 3). This LOD agrees with the values reported before with this type of tests [46,47] by using gold nanoparticles (8.5 × 10^5^ and 4.5 × 10^6^ EVs/µL), carbon black nanoparticles (9.2 × 10^6^ EVs/µL) or other commercial magnetic nanoparticles coated with polyacrylic acid (1.0 × 10^7^ EVs/µL). Figure 7c shows a representative example of the strips for standard EVs. Prompted by these results, this LFIA system was next tested with freshly isolated EV from human plasma and also directly with different volumes of plasma (Figure 7d). Quantification of isolated EVs using the optical calibration curve (Figure 7a) was compared to that obtained by NTA. Despite the different basis of these measuring principles, results were in the same range of concentration (3 × 10^9^ EVs/µL determined in LFIA vs. 7 × 10^9^ EVs/µL determined by NTA). Estimation of the number of EVs by NTA is based on the tracking of their Brownian displacements, whereas LFIA is a single step procedure, in which EVs are recognized by targeting their surface markers. Moreover, human plasma without previous treatment was used for the first time directly on the test strips. An excellent agreement was achieved for the number of EVs obtained in two different volumes using the calibration curve: 21.4 × 10^6^ EVs/µL in 1 µL and 20.9 × 10^6^ EVs/µL when a 2 µL plasma aliquot was tested. This is an indication of the absence of matrix effects. Thus, the magnetic LFIA developed in this work with Fe_3_O_4_@C nanoflowers was found suitable for analysis of complex samples such as plasma or serum with minimum sample preparation in a simple protocol.

In addition, another important strength of these particles is their rapid separation with a conventional magnet in 2 min (Figure 7e) while keeping their superparamagnetic behavior. Therefore, they show a great potential to easily enrich CD63+ EV for further functional or analytical assays.

## 4. Conclusions

Fe_3_O_4_@C nanoparticles were synthesized with the purpose of having the double function of separation and quantification at immunoassays. Their characteristics are summarized in Table 1. They exhibited superparamagnetic behavior, and their value of ζ potential confirms their stability in suspension.

These carbon coated nanoflowers were conjugated to neutravidin by using the carbodiimide chemistry and tested at lateral flow immunoassays with a biotin test line. The conjugations process was monitored by DLS and confirmed by LFIA based on biotin–neutravidin interaction line. Quantification was carried out by means of an optical reader and a magnetic sensor. With this basis, the nanoflowers were used to develop a lateral flow immunoassay for detection of EVs.

In summary, these Fe_3_O_4_@C magnetic particles proved to be effective for bioconjugation purposes due to the availability of carboxylic groups on their surface. Their carbon coating provided a strong optical signal when using them as labels in lateral flow assays. In fact, the LFIA developed in this work achieved lower LOD values than those obtained by means of other commercial magnetic nanoparticle labels for EV detection. Therefore, the Fe_3_O_4_@C magnetic particles employed in this study have a great potential of application as labels in EV enrichment and detection.

## Figures and Tables

**Table 1:** Characteristics of carbon-coated superparamagnetic oxide nanoflowers Fe_3_O_4_@C.

Carbon-Coated Superparamagnetic Oxide Nanoflowers (Fe_3_O_4_@C)	
Synthesis	One-pot solvothermal method
Composition (core/shell)	Magnetite cores Black carbon coating
Magnetite crystals size (d)	10–20 nm
Magnetization saturation	50 emu per g of magnetite
Mean size	129 nm
Mean hydrodynamic diameter	178 nm
Electrokinetic potential (ζ)	−32 mV
Rate magnetite/total mass	0.651%

## Supplementary Materials

The following are available online at https://www.mdpi.com/2079-6374/10/8/80/s1, Figure S1: (a) Photograph of the sensing planar coil. (b) Image of the device integrating the planar coil and the micropositioner. (c) 40 MHz Impedance variation measured in a scan of a sample of nanoparticles containing 32 μg of Fe_3_O_4_. (d) Impedance recording observed for the scan of an LFIA with 1 mg/mL of neutravidin in two consecutive scans in the sensor.

## References

[1] Colombo M., Carregal-Romero S., Casula M.F., Gutierrez L., Morales M.P., Boehm I.B., Heverhagen J.T., Prosperi D., Parak W.J. (2012). ChemInform abstract: Biological applications of magnetic nanoparticles. Chem. Soc. Rev..

[2] Zhang L., Dong W.-F., Sun H.-B. (2013). Multifunctional superparamagnetic iron oxide nanoparticles: Design, synthesis and biomedical photonic applications. Nanoscale.

[3] Knobel M., Nunes W.C., Socolovsky L.M., De Biasi E., Vargas J.M., Denardin J.C. (2008). Superparamagnetism and other magnetic features in granular materials: A review on ideal and real systems. J. Nanosci. Nanotechnol..

[4] Wu W., Wu Z., Yu T., Jiang C., Kim W.S. (2015). Recent progress on magnetic iron oxide nanoparticles: Synthesis, surface functional strategies and biomedical applications. Sci. Technol. Adv. Mater..

[5] McNamara K., Tofail S.A.M. (2017). Nanoparticles in biomedical applications. Adv. Phys. X.

[6] Salvador M., Moyano A., Martínez-García J.C., Blanco-López M.C., Rivas M. (2019). Synthesis of superparamagnetic iron oxide nanoparticles: SWOT analysis towards their conjugation to biomolecules for molecular recognition applications. J. Nanosci. Nanotechnol..

[7] Rafiee E., Khodayari M. (2016). Starch as a green source for Fe_3_O_4_@carbon core-shell nanoparticles synthesis: A support for 12-tungstophosphoric acid, synthesis, characterization, and application as an efficient catalyst. Res. Chem. Intermed..

[8] Mendes R.G., Koch B., Bachmatiuk A., El-Gendy A.A., Krupskaya Y., Springer A., Klingeler R., Schmidt O., Büchner B., Sanchez S. (2014). Synthesis and toxicity characterization of carbon coated iron oxide nanoparticles with highly defined size distributions. Biochim. Biophys. Acta Gen. Subj..

[9] Prajapat C.L., Sharma P., Gonal M.R., Vatsa R.K., Singh M.R., Ravikumar G. (2016). Synthesis and magnetic study of carbon coated iron oxide nanoparticles by laser ablation in solution. AIP Conf. Proc..

[10] Taylor A., Krupskaya Y., Costa S., Oswald S., Kramer K., Füssel S., Klingeler R., Büchner B., Borowiak-Palen E., Wirth M.P. (2010). Functionalization of carbon encapsulated iron nanoparticles. J. Nanoparticle Res..

[11] Bonanni A., Ambrosi A., Pumera M. (2012). On oxygen-containing groups in chemically modified graphenes. Chem. A Eur. J..

[12] Zhang X., Zhao F., Sun Y., Mi T., Wang L., Li Q., Li J., Ma W., Liu W., Zuo J. (2020). Development of a highly sensitive lateral flow immunoassay based on receptor-antibody-amorphous carbon nanoparticles to detect 22 β-lactams in milk. Sens. Actuators B Chem..

[13] Guoa J., Chenb S., Jinhong Guoa J., Ma X. (2020). Nanomaterial Labels in lateral flow immunoassays for point-of-care-testing. J. Mater. Sci. Technol..

[14] Zhang X., Yu X., Wen K., Li C., Mujtaba Mari G., Jiang H., Shi W., Shen J., Wang Z. (2017). Multiplex lateral flow immunoassays based on amorphous carbon nanoparticles for detecting three fusarium mycotoxins in maize. J. Agric. Food Chem..

[15] Liu B., Wang L., Tong B., Zhang Y., Sheng W., Pan M., Wang S. (2016). Development and comparison of immunochromatographic strips with three nanomaterial labels: Colloidal gold, nanogold-polyaniline-nanogold microspheres (GPGs) and colloidal carbon for visual detection of salbutamol. Biosens. Bioelectron..

[16] Rocha-Santos T.A.P. (2014). Sensors and biosensors based on magnetic nanoparticles. TrAC Trends Anal. Chem..

[17] Hervás M., López M.Á., Escarpa A. (2010). Simplified calibration and analysis on screen-printed disposable platforms for electrochemical magnetic bead-based inmunosensing of zearalenone in baby food samples. Biosens. Bioelectron..

[18] Yang Z., Zhang C., Zhang J., Bai W. (2014). Potentiometric glucose biosensor based on core-shell Fe_3_O_4_-enzyme-polypyrrole nanoparticles. Biosens. Bioelectron..

[19] Yola M.L., Eren T., Atar N. (2014). A novel and sensitive electrochemical DNA biosensor based on Fe@Au nanoparticles decorated graphene oxide. Electrochim. Acta.

[20] Wang J., Sun Y., Wang L., Zhu X., Zhang H., Song D. (2010). Surface plasmon resonance biosensor based on Fe_3_O_4_/Au nanocomposites. Colloids Surf. B Biointerfaces.

[21] Wang Y., Dostalek J., Knoll W. (2011). Magnetic nanoparticle-enhanced biosensor based on grating-coupled surface plasmon resonance. Anal. Chem..

[22] Li D., Wang J., Wang R., Li Y., Abi-Ghanem D., Berghman L., Hargis B., Lu H. (2011). A nanobeads amplified QCM immunosensor for the detection of avian influenza virus H5N1. Biosens. Bioelectron..

[23] Gan N., Wang L., Li T., Sang W., Hu F., Cao Y. (2013). A Novel signal-amplified immunoassay for myoglobin using magnetic core-shell Fe_3_O_4_@Au-multi walled carbon nanotubes composites as labels based on one piezoelectric sensor. Integr. Ferroelectr..

[24] Haun J.B., Yoon T.J., Lee H., Weissleder R. (2010). Magnetic nanoparticle biosensors. Wiley Interdiscip. Rev. Nanomed. Nanobiotechnol..

[25] Srinivasan B., Li Y., Jing Y., Xing C., Slaton J., Wang J.P. (2011). A three-layer competition-based giant magnetoresistive assay for direct quantification of endoglin from human urine. Anal. Chem..

[26] Zu P., Chan C.C., Koh G.W., Lew W.S., Jin Y., Liew H.F., Wong W.C., Dong X. (2014). Enhancement of the sensitivity of magneto-optical fiber sensor by magnifying the birefringence of magnetic fluid film with Loyt-Sagnac interferometer. Sens. Actuators B Chem..

[27] Hathaway H.J., Butler K.S., Adolphi N.L., Lovato D.M., Belfon R., Fegan D., Monson T.C., Trujillo J.E., Tessier T.E., Bryant H.C. (2011). Detection of breast cancer cells using targeted magnetic nanoparticles and ultra-sensitive magnetic field sensors. Breast Cancer Res..

[28] Sajid M., Kawde A.N., Daud M. (2015). Designs, formats and applications of lateral flow assay: A literature review. J. Saudi Chem. Soc..

[29] Huang X., Aguilar Z.P., Xu H., Lai W., Xiong Y. (2015). Membrane-based lateral flow immunochromatographic strip with nanoparticles as reporters for detection: A review. Biosens. Bioelectron..

[30] Moyano A., Serrano-pertierra E., Salvador M., Martínez-garcía J.C., Rivas M., Blanco-López M.C. (2020). Magnetic lateral flow immunoassays. Diagnostics.

[31] Panferov V.G., Safenkova I.V., Zherdev A.V., Dzantiev B.B. (2017). Setting up the cut-off level of a sensitive barcode lateral flow assay with magnetic nanoparticles. Talanta.

[32] Liu C., Jia Q., Yang C., Qiao R., Jing L., Wang L., Xu C., Gao M. (2011). Lateral flow immunochromatographic assay for sensitive pesticide detection by using Fe_3_O_4_ nanoparticle aggregates as color reagents. Anal. Chem..

[33] Wu J., Dong M., Zhang C., Wang Y., Xie M., Chen Y. (2017). Magnetic lateral flow strip for the detection of cocaine in urine by naked eyes and smart phone camera. Sensors.

[34] Workman S., Wells S.K., Pau C.P., Owen S.M., Dong X.F., LaBorde R., Granade T.C. (2009). Rapid detection of HIV-1 p24 antigen using magnetic immuno-chromatography (MICT). J. Virol. Methods.

[35] Zheng C., Wang X., Lu Y., Liu Y. (2012). Rapid detection of fish major allergen parvalbumin using superparamagnetic nanoparticle-based lateral flow immunoassay. Food Control.

[36] Wang D.B., Tian B., Zhang Z.P., Deng J.Y., Cui Z.Q., Yang R.F., Wang X.Y., Wei H.P., Zhang X.E. (2013). Rapid detection of Bacillus anthracis spores using a super-paramagnetic lateral-flow immunological detectionsystem. Biosens. Bioelectron..

[37] Wang D.B., Tian B., Zhang Z.P., Wang X.Y., Fleming J., Bi L.J., Yang R.F., Zhang X.E. (2015). Detection of bacillus anthracis spores by super-paramagnetic lateral-flow immunoassays based on “Road Closure”. Biosens. Bioelectron..

[38] Sharma A., Tok A.I.Y., Lee C., Ganapathy R., Alagappan P., Liedberg B. (2019). Magnetic field assisted preconcentration of biomolecules for lateral flow assaying. Sens. Actuators B Chem..

[39] Razo S.C., Panferov V.G., Safenkova I.V., Varitsev Y.A., Zherdev A.V., Dzantiev B.B. (2018). Double-enhanced lateral flow immunoassay for potato virus X based on a combination of magnetic and gold nanoparticles. Anal. Chim. Acta.

[40] Huang Z., Xiong Z., Chen Y., Hu S., Lai W. (2019). Sensitive and matrix-tolerant lateral flow immunoassay based on fluorescent magnetic nanobeads for the detection of clenbuterol in swine urine. J. Agric. Food Chem..

[41] Huang Z., Hu S., Xiong Y., Wei H., Xu H., Duan H., Lai W. (2019). Application and development of superparamagnetic nanoparticles in sample pretreatment and immunochromatographic assay. TrAC Trends Anal. Chem..

[42] Lago-Cachón D., Oliveira-Rodríguez M., Rivas M., Blanco-López M.C., Martínez-García J.C., Moyano A., Salvador M., García J.A. (2017). Scanning magneto-inductive sensor for quantitative assay of prostate-specific antigen. IEEE Magn. Lett..

[43] Moyano A., Salvador M., Martínez-García J.C., Socoliuc V., Vékás L., Peddis D., Alvarez M.A., Fernández M., Rivas M., Blanco-López M.C. (2019). Magnetic immunochromatographic test for histamine detection in wine. Anal. Bioanal. Chem..

[44] Lago-Cachón D., Rivas M., Martínez-García J.C., García J.A. (2013). Cu impedance-based detection of superparamagnetic nanoparticles. Nanotechnology.

[45] Rivas M., Lago-Cachón D., Martínez-García J.C., García J.A., Calleja A.J. (2014). Eddy-current sensing of superparamagnetic nanoparticles with spiral-like copper circuits. Sens. Actuators A Phys..

[46] Oliveira-Rodríguez M., López-Cobo S., Reyburn H.T., Costa-García A., López-Martín S., Yáñez-Mó M., Cernuda-Morollón E., Paschen A., Valés-Gómez M., Blanco-López M.C. (2016). Development of a rapid lateral flow immunoassay test for detection of exosomes previously enriched from cell culture medium and body fluids. J. Extracell. Vesicles.

[47] Oliveira-Rodríguez M., Serrano-Pertierra E., García A.C., Martín S.L., Mo M.Y., Cernuda-Morollón E., Blanco-López M.C. (2017). Point-of-care detection of extracellular vesicles: Sensitivity optimization and multiple-target detection. Biosens. Bioelectron..

[48] Wang H., Sun Y.B., Chen Q.W., Yu Y.F., Cheng K. (2010). Synthesis of carbon-encapsulated superparamagnetic colloidal nanoparticles with magnetic-responsive photonic crystal property. Dalton Trans..

[49] Nakagiri N., Manghnani M.H., Ming L.C., Kimura S. (1986). Crystal structure of magnetite under pressure. Phys. Chem. Miner..

[50] Zsigmondy R., Scherrer P. (1912). Bestimmung der inneren struktur und der größe von kolloidteilchen mittels röntgenstrahlen. Kolloidchemie Ein Lehrbuch.

[51] Wang L., Bao J., Wang L., Zhang F., Li Y. (2006). One-pot synthesis and bioapplication of amine-functionalized magnetite nanoparticles and hollow nanospheres. Chem. A Eur. J..

[52] Lakshmipriya T., Gopinath S.C.B., Tang T.H. (2016). Biotin-streptavidin competition mediates sensitive detection of biomolecules in enzyme linked immunosorbent assay. PLoS ONE.

[53] Lin Z., Wang X., Li Z.J., Ren S.Q., Chen G.N., Ying X.T., Lin J.M. (2008). Development of a sensitive, rapid, biotin-streptavidin based chemiluminescent enzyme immunoassay for human thyroid stimulating hormone. Talanta.

[54] Sai N., Chen Y., Liu N., Yu G., Su P., Feng Y., Zhou Z., Liu X., Zhou H., Gao Z. (2010). A sensitive immunoassay based on direct hapten coated format and biotin-streptavidin system for the detection of chloramphenicol. Talanta.

[55] Liu N., Nie D., Zhao Z., Meng X., Wu A. (2015). Ultrasensitive Immunoassays Based on Biotin-Streptavidin Amplified System for Quantitative Determination of Family Zearalenones.

[56] Liu R., Liu J., Xie L., Wang M., Luo J., Cai X. (2010). A fast and sensitive enzyme immunoassay for brain natriuretic peptide based on micro-magnetic probes strategy. Talanta.

[57] Kim D.S., Kim Y.T., Hong S.B., Kim J., Huh N.S., Lee M.K., Lee S.J., Kim B., Kim I.S., Huh Y.S. (2016). Development of lateral flow assay based on size-controlled gold nanoparticles for detection of hepatitis B surface antigen. Sensors.

[58] Thobhani S., Attree S., Boyd R., Kumarswami N., Noble J., Szymanski M., Porter R.A. (2010). Bioconjugation and characterisation of gold colloid-labelled proteins. J. Immunol. Methods.

