# Supplementary material for: Carbon-Coated Superparamagnetic Nanoflowers for Biosensors Based on Lateral Flow Immunoassays
Source: Biosensors (Basel). 2020 Jul 22;10(8):80. doi: 10.3390/bios10080080 (PMC7460469; doi:10.3390/bios10080080)
Supplement: Supplementary file 1 [file biosensors-10-00080-s001.pdf]

biosensors

MDPI

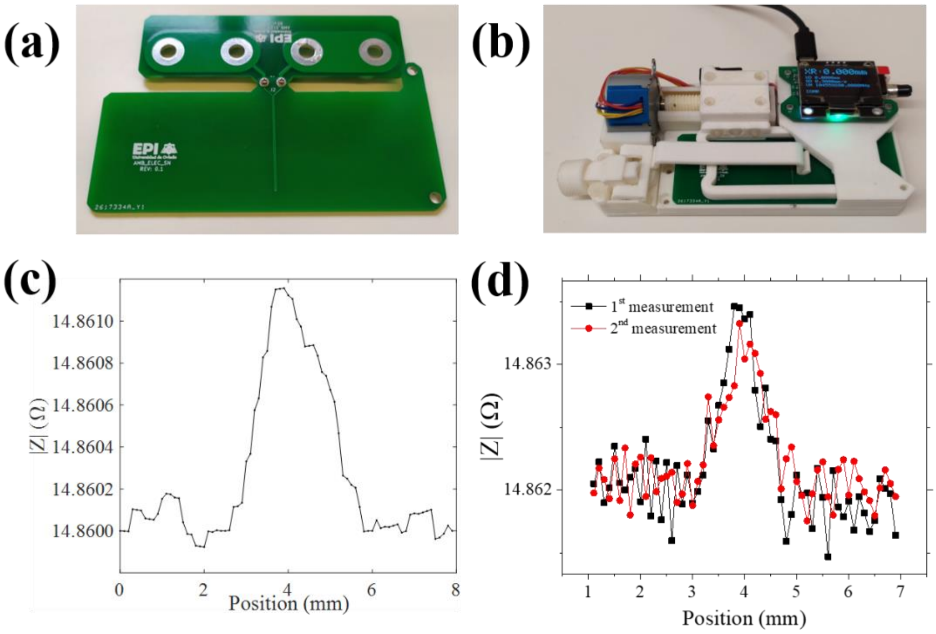

Figure S1. (a) Photograph of the sensing planar coil. (b) Image of the device integrating the planar coil and the micropositioner. (c) 40 MHz Impedance variation measured in a scan of a sample of nanoparticles containing 32 μg of Fe_3O_4. (d) Impedance recording observed for the scan of an LFIA with 1 mg/mL of neutravidin in two consecutive scans in the sensor.

An inductive sensor specifically developed to measure lateral flow strips was used to evaluate the magnetic LFIA. The transducer of the sensor consists of a planar coil with the shape of a double copper line that is printed on an insulating substrate. The coil is fed with a low amplitude radio frequency current flow while its impedance is continuously monitored by a precision four-point autobalancing impedance analyzer (Agilent 4294A) using 16048G test leads, 500 mV and 20–110 MHz excitation voltage (Supplementary Figure S1a).

For quantification purposes, we slide the LFIA in smooth contact over the planar coil. To do this, we use a micropositioner that was specifically designed for this application (Supplementary Figure S1b). Most components (all except the stepper motor) were 3D-printed using a PLA filament to avoid moving metallic parts that may induce spurious signals. The duration of one LFIA measurement is about 5 minutes.

The planar coil detects the varying magnetic flux produced by the presence of the magnetic particles close to it. It produces a change in the electric impedance which is directly proportional to the frequency of the driving current, the initial magnetic permeability of the particles and the total volume of their magnetic cores. A working frequency of 40 MHz has been selected, which optimizes the signal to noise ratio.

The sensitivity and resolution of the detection not only depend on the features of the sensor (geometry, size, and electronics), but on the properties of the particles. To achieve a significant magnetic permeability at radio frequencies, superparamagnetism is essential. Supplementary Figure S1c shows the variation of the impedance produced by a sample of unmodified particles. To account for all the particles disregarding their distribution in the sample, the cumulative integral of the peak is calculated to yield a response value in Ω·mm.

For a fixed frequency and type of particles, there is a linear correlation between the measured change of impedance and the volume or mass of the particles (see Figure 2 in ref [1]). For the particles of this work the sensitivity, given as the percentage change of the impedance per unit mass, is 0.23% per mg of Fe_3O_4.

Supplementary Figure S1d shows the variation of the impedance for one of the LFIAs of this work (1 mg/mL of neutravidin).

biosensors

MDPI

References

1. Lago-Cachón, D.; Oliveira-Rodríguez, M.; Rivas, M.; Blanco-López, M.C.; Martínez-García, J.C.; Moyano, A.; Salvador, M.; García, J.A. Scanning Magneto-Inductive Sensor for Quantitative Assay of Prostate-Specific Antigen. *IEEE Magn. Lett.* **2017**, *8*.
